# Rapid Reduction in Proteinuria With the Initiation of Voclosporin in Lupus Nephritis: A Single-Center Experience

**DOI:** 10.7759/cureus.105557

**Published:** 2026-03-20

**Authors:** Homa Timlin, Johnson Ong, Duvuru Geetha, Mohamed G Atta, Ihab Kamel, Boyoung Ahn, Timothy Kaniecki

**Affiliations:** 1 Rheumatology, Johns Hopkins University School of Medicine, Baltimore, USA; 2 Rheumatology, Loma Linda University School of Medicine, Loma Linda, USA; 3 Nephrology, Johns Hopkins University School of Medicine, Baltimore, USA; 4 Radiology, Johns Hopkins University School of Medicine, Baltimore, USA; 5 Internal Medicine, Johns Hopkins University School of Medicine, Baltimore, USA

**Keywords:** calcineurin inhibitor, complete renal response, early proteinuria reduction, lupus nephritis, retrospective cohort study, systemic lupus erythematous, urine protein creatinine ratio (upcr), voclosporin

## Abstract

Background and purpose

Lupus nephritis (LN) is a severe, life-threatening complication of systemic lupus erythematosus (SLE). Reduction in proteinuria is strongly associated with long‑term renal outcomes and is a key endpoint in LN clinical trials. Early initiation of immunosuppressive therapy improves renal recovery, and a rapid decline in proteinuria correlates with achieving remission at 12 months. Voclosporin may cause a mild, early decline in estimated glomerular filtration rate (eGFR) due to hemodynamic effects, which typically stabilizes with continued therapy. This study evaluated the effect of voclosporin on early (≤12‑week) proteinuria reduction and its tolerability when used in combination with standard immunosuppressive therapy in patients with biopsy‑proven LN.

Methods

We conducted a retrospective cohort study using the institutional review board-approved Epic Lupus Database at Johns Hopkins Bayview Medical Center, Baltimore, MD, USA. Patients were included if they had biopsy‑confirmed LN and received voclosporin. Demographics, clinical characteristics, and immunosuppressive regimens were abstracted from electronic medical records. Relative changes in urine protein creatinine ratio (UPCR) from baseline to four, eight, and 12 weeks were calculated with corresponding 95% confidence intervals (CIs).

Results

Twenty‑five patients with biopsy‑proven LN were treated with voclosporin; all received concurrent hydroxychloroquine and tolerated voclosporin without discontinuation. Most patients were treated with mycophenolate or mycophenolic acid (92%) and corticosteroids (96%; median prednisone‑equivalent dose 15 mg/day). Relative reductions in UPCR from baseline were observed at four weeks (-0.27; 95% CI, -0.49 to -0.05), eight weeks (-0.51; 95% CI, -0.65 to -0.37), and 12 weeks (-0.57; 95% CI, -0.71 to -0.43). No statistically significant change in eGFR was observed over the 12‑week treatment period.

Conclusion

Initiation of voclosporin in combination with standard immunosuppressive therapy in biopsy‑proven LN was associated with early improvement in renal activity, as reflected by significant reductions in UPCR beginning at four weeks. To our knowledge, this represents the largest case series to date describing early real‑world outcomes with voclosporin in LN. Given that persistent proteinuria may indicate ongoing renal inflammation and/or chronic damage, these findings support consideration of voclosporin as a first‑line therapeutic option for active LN.

## Introduction

Among the myriad complications associated with systemic lupus erythematosus (SLE), lupus nephritis (LN) stands as a critical and potentially life-threatening complication [1]. Epidemiological estimates suggest that 30% to 60% of adults and up to 70% of children with SLE will experience LN [2]. Early reduction in proteinuria predicts lower risk of end-stage renal disease (ESRD) and preservation of estimated glomerular filtration rate (eGFR).

Research conducted by Hoover et al. [3] has shown that the risk of LN patients progressing to ESRD increases over time, at rates of 11%, 17%, and 22% at five, 10, and 15 years, respectively. In the United States, patients of Black, Hispanic, and Asian ethnic descent exhibit higher rates of progression to ESRD compared to patients of Caucasian descent [4,5]. This disparity is thought to be multifactorial, including genetic predispositions or socioeconomic influences [3,6].

Patients with LN who achieve remission of clinical laboratory abnormalities, including creatinine and proteinuria levels, experience a significant improvement in long-term survival rates [7]. Rapid reduction in proteinuria within eight weeks is associated with a sustained response to therapy in the long term [8]. While the conventional 24-hour urine protein collection remains the gold standard for assessing proteinuria, the urine protein creatinine ratio (UPCR) is widely accepted as a reliable predictor of outcomes. Leung et al. [9] have concluded that UPCR measured in untimed urine specimens is a dependable measure of proteinuria in LN patients.

The updated American College of Rheumatology guideline has shifted toward UPCR ≤ 0.5 g/g for the proteinuria threshold for defining complete renal response in clinical decision-making [10]. In contrast, in 2012, the American College of Rheumatology recommended a more stringent UPCR < 0.2 g/g as part of the criteria for a complete renal response to treatment [11].

The aim of this study was to evaluate the extent of proteinuria reduction by UPCR, tolerability, and changes in eGFR following the initiation of voclosporin therapy when used with background immunosuppressive therapy in patients with biopsy-proven LN and persistent proteinuria greater than 0.2 g/g.

## Materials and methods

Patient selection

The patient cohort was identified through the Epic Lupus Database and approved by the institutional review board of Johns Hopkins Bayview Medical Center, Baltimore, MD, USA. A meticulous examination of their medical records was conducted to ensure comprehensive data retrieval. Patient data were retrospectively gathered from electronic medical records, which encompassed patient demographics, clinical manifestations, treatment regimens, and laboratory results.

Inclusion criteria for subjects were twofold: 1. Confirmation of LN, established through kidney biopsy, encompassing classes I through V of the disease, and 2. Initiation of voclosporin therapy after LN diagnosis.

It's worth noting that many patients received concurrent treatment with mycophenolate mofetil and corticosteroids; however, the absence of any of these treatments did not preclude inclusion in the study population.

Patients were excluded if they had reported non-compliance with voclosporin therapy, did not obtain any follow-up laboratory data within the initial 12-week treatment period, or had documented proteinuria < 0.2 g/g at the initiation of voclosporin.

Data collection

Measurements of patients' UPCR and eGFR prior to the initiation of voclosporin therapy were collected, which were established as baseline values. Assessments of UPCR and eGFR were obtained at approximately four-, eight-, and 12-week intervals from initiation of voclosporin. Additionally, an evaluation of various baseline lupus markers was obtained. Variables of interest included serologies (anti-Ro52, Ro60, La, Sm, and dsDNA antibodies), serum albumin levels, and the presence of hypocomplementemia (C3 and/or C4).

Statistical methods

Relative change in UPCR from baseline was calculated at four, eight, and 12 weeks following initiation of voclosporin. Statistical significance of the relative change from baseline was assessed using one-sample t-tests comparing the mean relative change to 0. Results are presented as mean ± standard deviation with corresponding 95% confidence intervals (CIs). A two-sided p-value < 0.05 was considered statistically significant. Statistical analyses were performed using Microsoft Excel (Microsoft Corp., Redmond, WA, USA).

Voclosporin protocol

All patients were started on voclosporin at 15.8 mg twice daily and quickly titrated up to 23.7 mg twice daily within a few days, as tolerated. Dose reductions to 15.8 mg twice daily were permitted in settings of abnormal renal function, defined as an eGFR of less than 60 mL/min/1.73 m².

## Results

Patient characteristics 

A total of 33 patients were initially identified as meeting inclusion criteria; four were excluded due to baseline proteinuria < 0.2 g/g, and four were excluded due to lack of follow-up laboratory data. In total, 25 patients were included in the final analysis. Demographic characteristics of the patients are summarized in Table 1. The mean age was 39.9 years (SD 12.0), and the mean age at SLE diagnosis was 33.4 years (SD 10.6). Most patients identified as Black (18/25, 72%), followed by Asian (3/25, 12%). All participants were female (25/25, 100%). Class V LN was most common (12/25, 48%), followed by class III (11/25, 44%).

**Table 1:** Demographics, Serologic Phenotyping, and Active Medication History in Patients with Systemic Lupus Erythematosus Treated with Voclosporin (N = 25) Lupus nephritis classes were not mutually exclusive; some patients had mixed proliferative and membranous disease. Data are presented as numbers (N) and percentages (%) unless otherwise specified. Continuous variables are presented as mean ± standard deviation (SD). Abbreviations: SD, standard deviation; LN, lupus nephritis; dsDNA, double-stranded DNA; SS-A, Sjögren’s syndrome-related antigen A; Ro52/Ro60, ribonucleoprotein antigens; C3, complement component 3; C4, complement component 4; ACE, angiotensin-converting enzyme; ARB, angiotensin receptor blocker; eGFR, estimated glomerular filtration rate; UPCR, urine protein creatinine ratio.

Characteristic	Value
Female Sex, N (%)	25 (100.0)
Age, Mean (SD)	39.9 (12.0)
Age at Diagnosis, Mean (SD)	33.4 (10.6)
Race	
Asian, N (%)	3 (12.0)
Black, N (%)	18 (72.0)
Caucasian, N (%)	2 (8.0)
Hispanic, N (%)	2 (8.0)
Active Medications	
Corticosteroids, N (%)	24 (96.0)
Prednisone Equivalent Dose (mg), Median	15
Prednisone Equivalent Dose (mg), Mean (SD)	16.1 (10.7)
Hydroxychloroquine, N (%)	25 (100.0)
Azathioprine, N (%)	1 (4.0)
Mycophenolate, N (%)	23 (92.0)
Belimumab, N (%)	6 (24.0)
History of Prior Calcineurin Inhibitor, N (%)	5 (20.0)
ACE Inhibitor or ARB, N (%)	19 (76.0)
Lupus Nephritis Class	
I, N (%)	1 (4.0)
II, N (%)	1 (4.0)
III, N (%)	11 (44.0)
IV, N (%)	6 (24.0)
V, N (%)	12 (48.0)
Auto-Antibodies	
dsDNA, N (%)	19 (76.0)
Sm, N (%)	18 (72.0)
SS-A (total Ro), N (%)	19 (76.0)
Ro52, N (%)	6 (24.0)
Ro60, N (%)	13 (52.0)
Baseline Albumin Level (g/dL), Mean (SD)	3.8 (0.6)
Active C3 Hypocomplementemia, N (%)	6 (24.0)
Active C4 Hypocomplementemia, N (%)	5 (20.0)
Baseline Glomerular Filtration Rate, Mean (SD)	96.0 (21.7)
Baseline Urine Protein: Creatinine Ratio Range, Ratio (Mean)	0.201-8.257 (1.635)

Medication dosing is summarized in Table 2. All patients (25/25, 100%) were receiving hydroxychloroquine, and 24 out of 25 patients (96%) were receiving corticosteroids at the time of voclosporin initiation. The mean corticosteroid dose was 16.1 mg prednisone-equivalent daily (SD 10.7), with a median dose of 15 mg daily. The majority of patients (23/25, 92%) were receiving concurrent mycophenolate therapy. Nineteen of 25 patients (76%) were also receiving angiotensin-converting enzyme inhibitor or angiotensin receptor blocker therapies. No patients (0/25, 0%) were treated with sodium-glucose cotransporter-2 inhibitors. Five of 25 patients (20%) had a history of prior calcineurin inhibitor exposure.

**Table 2:** Immunosuppressive Medication Dosing at the Time of Voclosporin Initiation Data are presented as numbers (N) and percentages (%).

Hydroxychloroquine (Total N = 25)	
200 mg daily, N (%)	4 (16.0)
300 mg daily, N (%)	6 (24.0)
400 mg daily, N (%)	15 (60.0)
Mycophenolate (Total N = 23)	
2000 mg total daily dose, N (%)	6 (26.1)
3000 mg total daily dose, N (%)	17 (73.9)
Azathioprine (Total N = 1)	
1.5 mg/kg daily, N (%)	1 (100.0)
Belimumab (Total N = 6)	
200 mg weekly, N (%)	6 (100.0)

Baseline laboratory data are summarized in Table 1. The mean eGFR at baseline was 96.0 mL/min/1.73 m² (SD 21.7), indicating relatively preserved renal function. Baseline UPCR at the time of voclosporin initiation ranged from 0.201 to 8.257 g/g, with a mean of 1.635 g/g.

Complete renal response 

**Figure 1:**
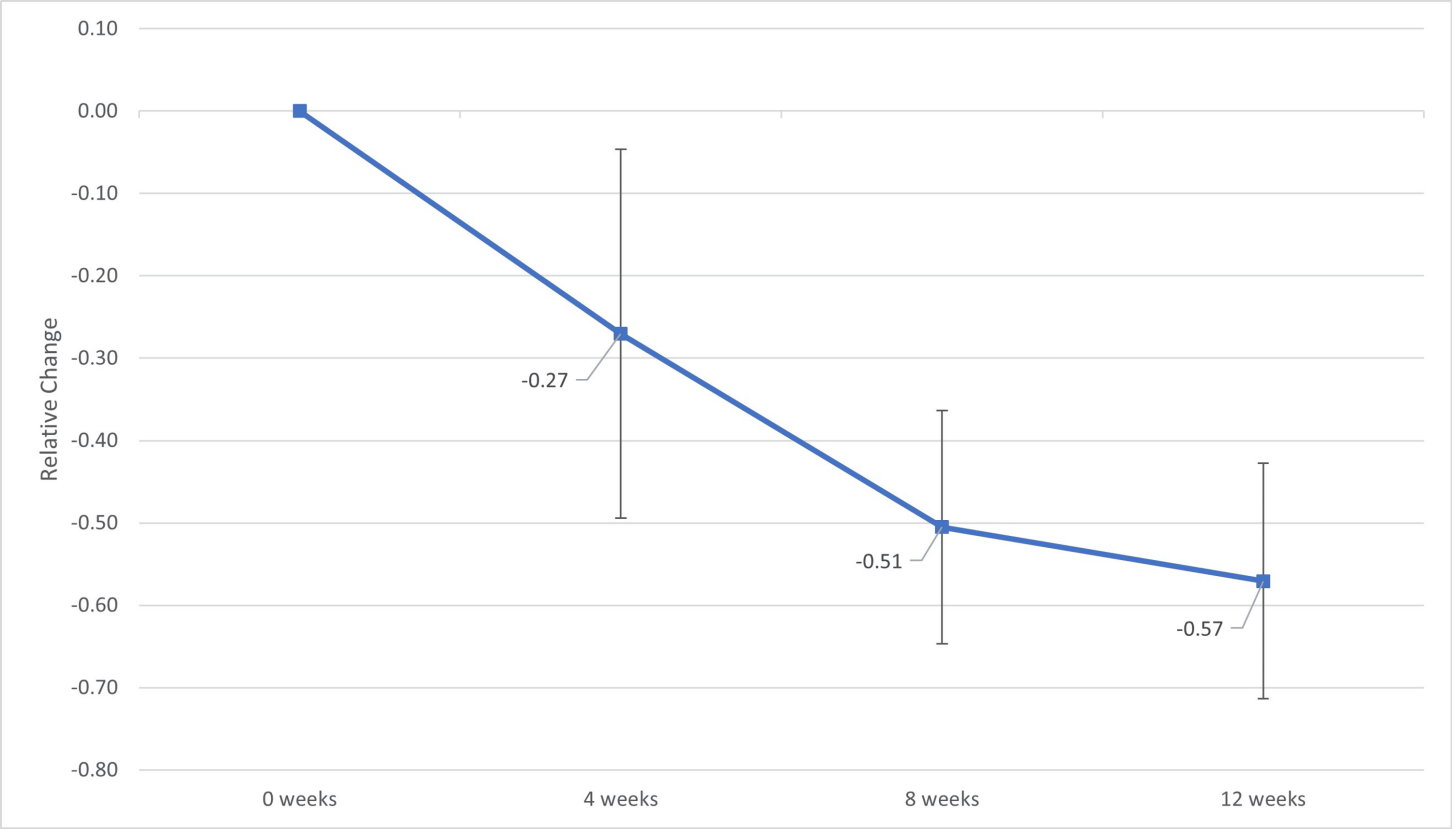
Relative Change in Urine Protein Creatinine Ratio (UPCR) From Baseline at Four, Eight, and 12 Weeks Following Initiation of Voclosporin Therapy Mean relative change in UPCR from baseline at four, eight, and 12 weeks following initiation of voclosporin therapy in patients with biopsy-proven lupus nephritis. Data are presented as mean ± standard deviation. Error bars represent 95% confidence intervals. Statistical significance of relative change from baseline was assessed using one-sample t-tests. A two-sided p-value <0.05 was considered statistically significant.

Given the wide baseline range of UPCR, relative changes were calculated as part of this analysis. Trends are shown in Figure 1. There was a statistically significant relative reduction in UPCR at four weeks following initiation of voclosporin (N = 25; mean relative change −0.27; 95% CI, −0.51 to −0.03; t(24) = −2.37, p = 0.03). This effect increased at eight weeks (N = 24; mean relative change −0.51; 95% CI, −0.66 to −0.36; t(23) = −7.00, p < 0.001) and persisted at 12 weeks (N = 22; mean relative change −0.57; 95% CI, −0.72 to −0.42; t(21) = −7.82, p < 0.001) compared to baseline.

**Figure 2:**
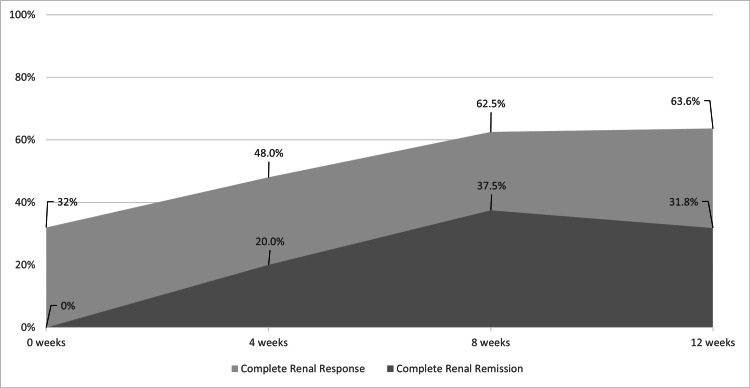
Percentage of Patients Achieving Complete Renal Response and Complete Renal Remission at 0, Four, Eight, and 12 Weeks Following Initiation of Voclosporin Add-On Therapy (N = 25). Data are presented as numbers (N) and percentages (%).

At 12 weeks, 14 out of 22 patients (63.6%) achieved complete renal response (UPCR ≤ 0.5 g/g). Of the total cohort, seven out of 25 patients (28.0%) achieved complete renal remission (UPCR < 0.2 g/g) by 12 weeks of therapy, as shown in Figure 2. The mean relative change in eGFR at weeks four, eight, and 12 did not reach statistical significance. The mean eGFR at 12 weeks was 93.4 mL/min/1.73 m².

**Figure 3:**
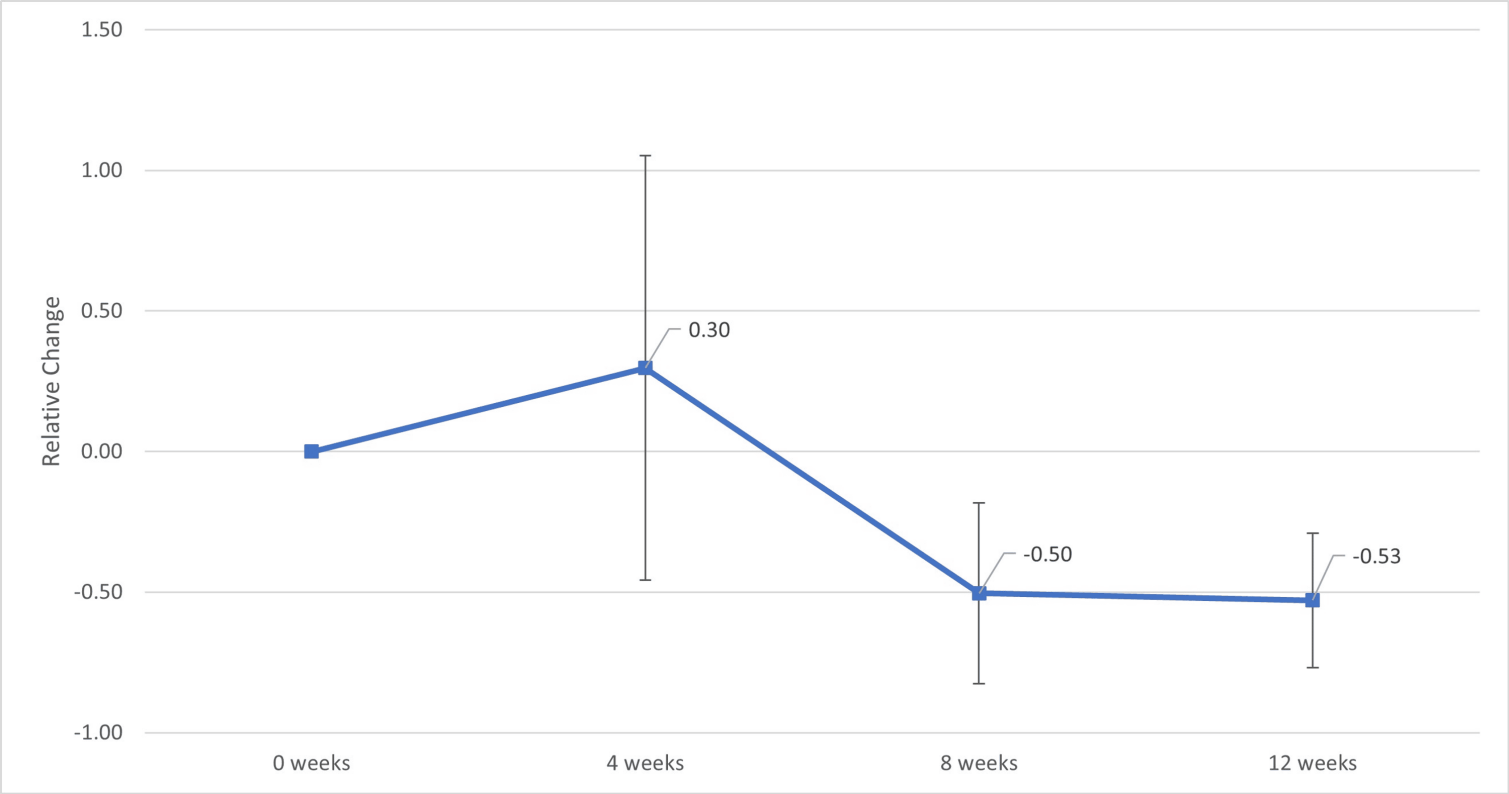
Relative Change in Urine Protein Creatinine Ratio (UPCR) from Baseline (0 weeks) in Patients Previously on Tacrolimus with Proteinuria and Lupus Nephritis After Starting Voclosporin Relative change in UPCR from baseline (0 weeks) at four, eight, and 12 weeks following initiation of voclosporin in patients previously treated with tacrolimus (N = 5). Data are presented as mean ± standard deviation. Error bars represent 95% confidence intervals. Statistical significance of relative change from baseline was assessed using one-sample t-tests. A two-sided p-value <0.05 was considered statistically significant.

**Figure 4:**
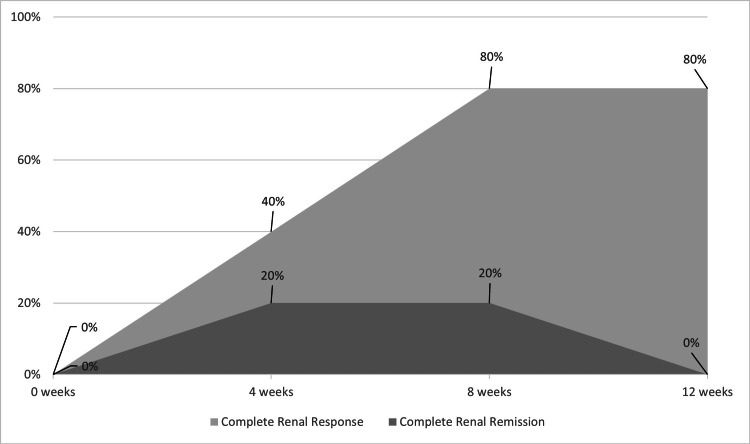
Percentage of Patients Previously Treated With Tacrolimus Achieving Complete Renal Response and Complete Renal Remission at 0, Four, Eight, and 12 Weeks Following Initiation of Voclosporin Add-On Therapy (N = 5). Data are presented as numbers (N) and percentages (%).

In the subgroup of patients previously treated with tacrolimus prior to transitioning to voclosporin (n = 5), similar trends were observed (Figures 3, 4). At four weeks, the relative change in UPCR was not statistically significant (mean 0.30; 95% CI, −0.77 to 1.37; t(4) = 0.77, p = 0.48). However, a statistically significant reduction was observed at eight weeks (mean −0.50; 95% CI, −0.96 to −0.04; t(4) = −3.07, p = 0.04), which persisted at 12 weeks (mean −0.53; 95% CI, −0.87 to −0.19; t(4) = −4.33, p = 0.01). All five out of five patients (100%) had ongoing proteinuria at the start of voclosporin therapy. By 12 weeks, four out of five patients (80%) achieved complete renal response (UPCR ≤ 0.5 g/g).

## Discussion

LN remains a formidable complication of SLE, requiring early detection and proactive intervention. The last decade has seen significant advancements in therapeutic options, with voclosporin, belimumab, and obinutuzumab demonstrating favorable outcomes. Inflammation associated with LN contributes to progressive scarring and fibrosis, which may result in ESRD in up to 30% of affected patients [12]. Early response to immunosuppressive therapy has consistently been shown to predict favorable long-term renal outcomes [13,14].

In this study, 14 out of 22 patients (63.6%) achieved complete renal response by 12 weeks of voclosporin therapy. Of the total cohort, seven out of 25 patients (28%) achieved complete renal remission. Relative reduction in proteinuria from baseline was statistically significant by week 4. The AURORA-1 trial provided the pivotal 52-week efficacy data supporting voclosporin in LN [15]. Our findings extend these data by demonstrating early improvement in proteinuria within routine clinical practice. In contrast, Ginzler et al. [16] reported that 22.5% of patients receiving mycophenolate achieved complete remission within 24 weeks. While cross-trial comparisons must be interpreted cautiously, the early response observed in our cohort supports the additive benefit of voclosporin to background immunosuppressive therapy.

Prior studies of voclosporin reported a reduction in eGFR as the most frequently observed adverse event in AURA-LV and AURORA-1 [17]. These effects were generally mild and reversible. In the present study, no statistically significant change in eGFR was observed over 12 weeks of therapy. Furthermore, long-term data from AURORA-2 demonstrated sustained reductions in proteinuria, higher complete renal response rates, and stable renal function over three years compared with the control group [18].

Limitations

This study has several limitations. It was a retrospective, single-center analysis with a relatively small sample size, which may limit generalizability. Only 22 patients had complete 12-week UPCR data available for response analysis. There was no control group, and background immunosuppressive regimens and corticosteroid tapering were not standardized, which may confound the interpretation of treatment response. Additionally, follow-up was limited to 12 weeks, precluding assessment of long-term renal outcomes and safety. 

## Conclusions

The introduction of voclosporin, in combination with other immunosuppressive treatments, in patients diagnosed with biopsy-proven LN resulted in a significant improvement in UPCR (≤ 0.5 g/g), with observable improvements emerging as early as four weeks into treatment and a majority achieving a complete response by week 12 of treatment. This effect was seen even in patients with exposure to alternative calcineurin inhibitors (tacrolimus) who previously had not reached these levels of improvement in urine proteinuria. Moreover, patients under voclosporin treatment experienced relatively stable renal function, as demonstrated by eGFR measurements. These findings support the first-line incorporation of voclosporin into the therapeutic arsenal for managing active LN. This study is limited by a small sample size and a retrospective design. Moreover, further studies are needed to analyze long-term responses in routine clinical care.
